# Early indicators of problematic grief trajectories following bereavement

**DOI:** 10.1080/20008198.2018.1423825

**Published:** 2018-01-19

**Authors:** A. A. A. Manik J. Djelantik, Geert E. Smid, Rolf J. Kleber, Paul A. Boelen

**Affiliations:** ^a^ Department of Clinical Psychology, Utrecht University, Utrecht, The Netherlands; ^b^ Arq Psychotrauma Expert Group, Diemen, The Netherlands; ^c^ Foundation Centrum ’45, Diemen, The Netherlands

**Keywords:** Prolonged Grief Disorder, trajectory, grief, indicators, screening tool, PGD, loss, bereaved individual, Trastorno por duelo prolongado, Trayectoria, Indicadores, Instrumento de discriminación, TDP, perdida, persona en duelo, 延长哀伤障碍, 轨迹, 哀伤, 指标, 筛选工具, PGD, 丧失, 丧亲者, • We found two classes with a problematic grief trajectory in adults over the first two years after a loss.• The endorsement of symptoms ‘yearning’, ‘stunned’, ‘life is empty’ and ‘bitterness’ as present ‘often’ could act as early indicators of a problematic grief trajectory.

## Abstract

**Background**: Little is known about the development of Prolonged Grief Disorder (PGD) symptoms over time in adults. For clinical purposes, it would be useful to have knowledge about early indicators of a problematic grief trajectory.

**Objective**: This study aimed to identify classes of bereaved individuals with similar trajectories of PGD symptoms and to design a provisional screening tool including symptoms predicting membership of classes with problematic grief trajectories.

**Method**: In a Dutch sample of 166 bereaved individuals, we conducted a latent class analysis to identify classes of bereaved individuals with similar trajectories of PGD symptoms between two time points (mean of 6 and 18 months post-loss, respectively). Next, we used Receiver Operating Characteristic (ROC) analyses to examine which symptoms at baseline best predicted membership of classes with problematic grief trajectories.

**Results**: We found four different classes: a class including individuals with persistent high PGD symptoms (class 1, 6%), a class of individuals with persistent moderate PGD symptoms (class 2, 35%), a class of individuals with slightly decreasing moderate PGD symptoms (class 3, 33%) and a class of individuals with persistent low PGD symptoms (class 4, 26%). The endorsement of symptoms ‘yearning’, ‘stunned’, ‘life is empty’ and ‘bitterness’ as present ‘often’ during the preceding month at baseline best-predicted membership of class 1 or 2.

**Conclusions**: Two classes of individuals with problematic grief trajectories were identified. Four symptoms were found which could act as early indicators of these two classes in a provisional screening tool.

## Abstract (es)

**Contexto**: Se sabe poco sobre el desarrollo en adultos de síntomas a lo largo del tiempo del llamado Trastorno de Duelo Prolongado (TDP). Por propósitos clínicos, es de utilidad tener conocimiento de indicadores precoces de una trayectoria de duelo problemática.

**Objetivo**: Este estudio pretende identificar clases de individuos en duelo con trayectorias similares y diseñar un instrumento de discriminación provisional que incluya síntomas que predigan quiénes formaran parte de grupos con trayectorias de duelo problemáticas.

**Método**: En una muestra holandesa de 166 personas en duelo, condujimos un análisis de clases latente cara a identificar grupos de individuos en duelo con similares trayectorias y síntomas de TDP entre dos momentos diferentes (una media de 6 y 18 meses post pérdida, respectivamente). A continuación, aplicamos análisis de características operativas de receptor (ROC) para examinar qué síntomas basales predicen mejor formar parte de un grupo con trayectorias de duelo problemáticas.

**Resultados**: Encontramos 4 grupos diferentes: uno que incluye individuos con niveles altos y persistentes de síntomas de TDP (clase, 1.6%). Otro con síntomas persistentes moderados de TDP (clase, 2.35%), otro grupo con síntomas de TDP disminuyendo poco a poco (clase, 3.33%) y, finalmente, otro grupo con síntomas leves persistentes de TDP (clase, 4.26%). La presencia de síntomas como ‘añoranza’, ‘anonadamiento’, ‘la vida se ha vaciado de sentido’ y ‘amargura’ con presencia ‘frecuente’ durante el mes previo al momento basal, resultó ser el mejor predictor de pertenencia a las dos primeras clases más graves.

**Conclusiones**: Se identificaron dos grupos de personas con trayectorias de duelo complicadas. Se encontraron, con un instrumento de discriminación provisional, cuatro síntomas que pueden ser considerados como indicadores precoces de estos dos grupos.

## Abstract (zh)

**背景：**关于成人延长哀伤障碍（PGD）症状随时发展的发现较少。出于临床目的，了解问题哀伤轨迹的早期指标是有用的。

**目标：**本研究旨在识别具有相似 PGD 症状轨迹的丧亲者的不同类别，并设计一个临时筛选工具，包含了可以预测问题哀伤轨迹类别的症状。

**方法：**在166名荷兰籍的丧亲者样本中，我们进行了一个潜在类别分析来识别具有相似 PGD 症状轨迹的丧亲者的类别。两次测量时间点分别在丧亲后平均6个月和18个月。然后我们使用接受者特征分析（ROC）考察哪一个基线症状可以最好地预测问题哀伤轨迹的类别。

**结果：**我们发现四个不同的类别：一类包括了持续的高 PGD 症状（类别1，6%），一类包括了持续中等 PGD 症状（类别2，35%），一类包括了稍微减轻的中等 PGD 症状（类别3，33%），一类包括了持续的低 PGD 症状（类别4，26%）。在基线前一月中‘经常’出现‘怀念’‘震惊’‘生活空虚’和‘感到苦涩’症状可以最优预测类别1或者2。

**结论：**识别了两个问题哀伤轨迹类别。发现4个症状可以作为临时筛查工具中识别这两个类别的早期指标。

Experiencing some form of grief is universal in individuals who have lost a loved one. However, a significant minority of bereaved individuals will develop Prolonged Grief Disorder (PGD), i.e. persistent and debilitating grief reactions (Lundorff, Holmgren, Zachariae, Farver-Vestergaard, & O’Connor, 2017). Recently, PGD has been proposed for inclusion in the 11th edition of the International Statistical Classification of Diseases and Related Health Problems (Maercker et al., 2013; Prigerson et al., 2009). A slightly different conceptualization, named Persistent Complex Bereavement Disorder (PCBD), has been introduced in the 5^th^ Diagnostic and Statistical Manual of Mental Disorders as a disorder requiring further research (American Psychiatric Association, 2013). PGD and PCBD strongly overlap in terms of symptoms, prevalence and health correlates (Maciejewski, Maercker, Boelen, & Prigerson, 2016).

Little is known about the development of PGD symptoms over time. In two subsequent studies (Melhem, Porta, Shamseddeen, Walker Payne, & Brent, 2011; Melhem, Porta, Walker Payne, & Brent, 2013), children who had lost a parent due to suicide, accident or sudden natural death were followed up to 33 months after bereavement. Latent Class Growth Analysis (LCGA) was conducted to identify classes of children with similar grief trajectories. The researchers identified a class with high and sustained PGD symptoms (10%), a class with initially severe but rapidly declining PGD symptoms (31%) and a class with gradual decrease of PGD symptoms (59%). Next, the researchers identified a set of symptoms that were accurately related with membership of the high and sustained class using Receiver Operating Characteristic (ROC) analyses. This set included the following symptoms: ‘Longing and yearning for the deceased’, ‘inability to accept the death’, ‘shock’, ‘disbelief’, ‘loneliness’ and ‘a changed worldview’. These items were subsequently put forth as items in a screening tool for disturbed grief in children.

To our knowledge, classes including individuals with different trajectories of PGD symptoms have not yet been examined among adults. It is also largely unclear what symptoms in the first year following loss predict a pervasive trajectory. Such knowledge could help caregivers in their decisions regarding referrals for more extensive diagnostic evaluation and/or follow-up visits. This will make care for bereaved individuals more effective in terms of costs and organization.

The current study sought to extend existing knowledge on the development of PGD symptoms and early indicators among bereaved adults. Similar to Melhem et al. (2013), we first aimed to identify classes of bereaved individuals based on their grief trajectory. We expected that we would identify at least three classes. Next, we used ROC analyses to identify early indicators of the classes with a pervasive grief trajectory.

## 1. Methods

### 1.1. Participants and procedure

Data were gathered in the context of a larger study about grief (e.g. Djelantik, Smid, Kleber, & Boelen, 2017). Professional and lay mental health care workers handed out questionnaires to bereaved individuals; in our study, we included data from 269 participants who were bereaved less than one year ago. Participants gave written informed consent. The research programme was approved by an ethical review board. A total of 166 participants completed the same questionnaires one year later and were included for further analyses

Most participants (*n* = 128; 77%) were women; 82 participants (49%) had been to college or university. Mean age of the participants was 54.5 (*SD* = 12.4) years. Losses were due to a natural cause in 150 (90%) cases and an unnatural cause (i.e. suicide, accident, homicide) in 16 (10%) cases. Twenty participants (12%) had lost a child, 103 (62%) a spouse/partner and 43 (26%) a loved one other than a partner or child (e.g. friend, parent, sibling). Losses occurred on average six (*SD* = 3.2) months before completion of the first measures (Time 1 = T1). Table 1 summarizes analyses comparing participants who filled in questionnaires only at T1 (drop-outs) and participants who completed questionnaires on both time points (completers). There were no significant differences between the two groups.

**Table 1.** Differences between the participants dropping out between T1 and T2 (drop-outs) and those who continued to participate (completers).

	Drop-out (*n* = 103)	Completers (*n* = 166)	Significant differences between the groups
Gender, *n* (%)			χ^2^(1, *n* = 269) = −0.94
Male	29 (28)	38 (23)	
Female	74 (72)	128 (77)	
Age (*SD*)	52.51 (13.98)	54.45 (12.43)	*t*(267) = −1.15
Education, *n* (%)			χ^2^(1, *n* = 269) = 0.09
Low level of education	54 (52)	84 (51)	
High level of education	49 (48)	82 (49)	
Violent cause, *n* (%)			χ^2^(1, *n* = 269) = 1.51
Yes	15 (15)	16 (10)	
No	88 (85)	150 (90)	
Kinship, *n* (%)			χ^2^(2, *n* = 269) = 1.77
Partner	56 (54)	103 (62)	
Child	13 (13)	20 (12)	
Someone other than partner/child	34 (33)	43 (26)	
Time since the loss in months (*SD*)	6.63 (3.48)	6.49 (3.28)	*t*(267) = 0.33
Mean total score of the PGD scale (*SD*)	29.18 (8.86)	27.86 (9.37)	*t*(266) = 1.14

PGD = Prolonged Grief Disorder; *SD* = Standard Deviation. There were no significant differences between the groups.

### 1.2. Measures

#### 1.2.1. Prolonged Grief Disorder scale

The PGD scale contains 11 items representing criteria for PGD as proposed by Prigerson et al. (2009). The endorsement of symptoms in the last month is rated on a Likert scale (5-point scale with anchors 1 = never to 5 = always). In the current sample, Cronbach’s α was .91 at T1 and .93 at T2.

### 1.3. Statistical analyses

First, we conducted Latent Class Analysis (LCA) using MPlus version 7.3.1 (Muthén & Muthén, 1998–2011) to identify classes of trajectories of PGD symptoms using the sum-score of the PGD-scale at T1 and T2. We started with a 1-class model and then increased the number of classes until we reached the best-fitting model using goodness-of-fit statistics.

Next, we conducted ROC analyses for each of the 11 symptoms to identify the early indicators of distress using SPSS version 23. We first considered the value of the Area Under the Curve (AUC). An AUC of more than 0.80 is an indication for a good diagnostic test (Cantor & Kattan, 2000). Second, we looked at the sensitivity and specificity of each distinct symptom. With regard to screening tools for mental disorders with a low prevalence in a population, like PGD, prior studies (i.e. Smits, Smit, Cuijpers, & De Graaf, 2007) indicated that sensitivity is more important than specificity. Most screeners for PTSD have at least a sensitivity level of 0.80 (Mouthaan, Sijbrandij, Reitsma, Gersons, & Olff, 2014). Accordingly, we used a sensitivity level of 0.80 as the lower limit to identify early indicators. Third, we attempted to construct a provisional screening tool with these indicators. Therefore, we conducted ROC analyses on the sum-score of the identified early indicators and we chose a cut-off score based on a sensitivity above 0.80 and the highest possible specificity level. Applying a screening tool with a sensitivity of 0.80 to a group of 200 persons, given a prevalence of 10% of the disorder of interest (Lundorff et al., 2017), will result in 16 individuals who would be correctly identified as individuals at risk for the disorder and four individuals not identified as such. Subsequently, we calculated the positive predictive value (PPV) and the negative predictive value (NPV) for this set of items.

In the LCA, the 0.52% missing values were handled by Full Information Maximum Likelihood (FIML). For the ROC analysis, the missing values were handled by list-wise deletion.

**Figure 1.**
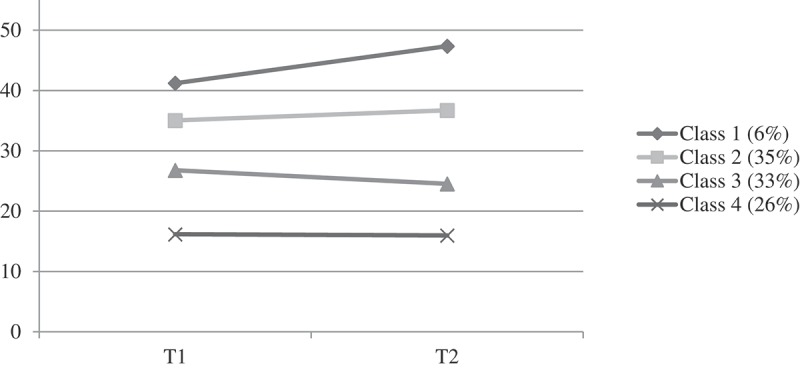
Estimated grief classes for the four-class solution. T1 = mean of six months after loss. T2 = mean of 18 months after loss.

## 2. Results

### 2.1. Latent class analysis

The fit indices for the latent class solutions are presented in Table 2. In the five-class solution, a very small sample size for one of the classes (*n* = 1) was found. Therefore, we did not consider the five-class solution and chose not to examine more classes. Among the remaining solutions, the four-class solution had the best combination of a low BIC (Bayesian Information Criterion), the lowest SS-BIC (Sample-Size Adjusted BIC), highest entropy and suitable sample sizes. The four-class solution included a class of individuals with persistent high PGD symptoms (class 1; 6%, intercept 41.21, slope 6.14, *p* = .10), a class of individuals with persistent moderate PGD symptoms (class 2; 35%, intercept 35.03, slope 1.67, *p* = .11), a class of individuals with decreasing moderate PGD symptoms (class 3; 33%, intercept 26.77, slope −2.25, *p* = .01) and a class of individuals with persistent low PGD symptoms (class 4; 26%, intercept 16.16, slope −0.19, *p* = .81) (see Figure 1).

**Table 2.** Goodness-of-fit statistics for 1 to 5 class solutions.

Classes	Log likelihood	AIC	BIC	SS-BIC	Entropy	BLRT	Smallest sample size (*n*)
1 class	−1233.824	2475.648	2488.096	2475.431			
2 class	−1170.968	2355.936	2377.720	2355.557	0.81	*p *= .00	80
3 class	−1153.859	2327.718	2358.837	2327.177	0.78	*p *= .00	47
4 class	−1146.935	2319.870	2360.326	2319.167	0.82	*p *= .00	9
5 class	−1138.358	2308.716	2358.508	2307.850	0.86	*p *= .00	1

AIC = Akaike Information Criterion; BIC  = Bayesian Information Criterion; SS-BIC = Sample Size Adjusted BIC; BLRT = bootstrapped likelihood ratio test.

### 2.2. ROC analysis

Two classes emerging from the LCA were considered to represent problematic grief trajectories, namely class 1 with individuals with a persistent high sum score and class 2 with a persistent moderate sum score. Therefore, we decided to run the analysis twice. First, we investigated the sensitivity and specificity of each item for inclusion in class 1, and secondly for inclusion in either class 1 or class 2.

With regard to class 1, the symptom items ‘yearning’, ‘stunned’, ‘life is empty’ and ‘bitterness’ scored with ≥ 4 could be selected (Table 3). With regard to the prediction of membership of class 1 or 2, there were no items with both an AUC above the 0.80 and sensitivity above 0.80 (Table 4). Therefore, we considered only the four symptoms we found in the first analysis as possible early indicators.

**Table 3.** Sensitivity and specificity of items with a score ≥ 4 at T1 (Mean: six months after loss) for a positive outcome for persistent high PGD symptoms (class 1).

Symptoms	Frequency of ≥ 4 scores at T1 (%)	Sens	Spec	AUC	*p*
Non-acceptance	19.3	0.556	0.825	0.562	.53
**Yearning**	**65.1**	**1.000**	**0.375**	**0.834**	.**00**
**Stunned**	**26.5**	**0.889**	**0.635**	**0.843**	.**00**
Mistrust	8.4	0.778	0.955	0.874	.00
**Life is empty**	**30.7**	**0.889**	**0.726**	**0.870**	.**00**
Numbness	15.1	0.778	0.885	0.871	.00
**Bitterness**	**22.3**	**0.889**	**0.815**	**0.858**	.**00**
Part of self died	34.3	0.778	0.682	0.835	.00
Functioning	22.3	0.556	0.796	0.705	.04
Difficulty moving on	25.3	0.667	0.771	0.797	.00
Avoidance	4.2	0.222	0.680	0.615	.25

Items in boldface type correspond to the items chosen for the screening tool. AUC = Area under the curve; Sens = sensitivity; Spec = specificity.

**Table 4.** Sensitivity and specificity of items with a score ≥ 4 at T1 (%) at T1 (Mean: six months after loss) for a positive outcome for a problematic grief trajectory (class 1 or 2).

Symptoms	Frequency of ≥ 4 scores at T1 (%)	Sens	Spec	AUC	*p*
Non-acceptance	19.3	0.412	0.958	0.756	.00
Yearning	65.1	0.882	0.495	0.789	.00
Stunned	26.5	0.536	0.927	0.840	.00
Mistrust	8.4	0.203	1.000	0.777	.00
Life is empty	30.7	0.600	0.906	0.851	.00
Numbness	15.1	0.300	0.958	0.859	.00
Bitterness	22.3	0.457	0.948	0.803	.00
Part of self died	34.3	0.614	0.854	0.815	.00
Functioning	22.3	0.400	0.906	0.750	.00
Difficulty moving on	25.3	0.486	0.917	0.846	.00
Avoidance	4.2	0.100	1.000	0.658	.00

AUC = Area under the curve; Sens = sensitivity; Spec = specificity.

**Table 5.** Provisional screening tool for problematic grief trajectories.

Early indicators for distress	Score (1 = never, 2 = rarely, 3 = sometimes, 4 = often, 5 = always)
I feel myself longing and yearning for the deceased	1-2-3-4-5
I feel stunned, dazed or shocked over his/her death	1-2-3-4-5
I feel that life is empty or meaningless without the deceased	1-2-3-4-5
I feel bitter over his/her death	1-2-3-4-5
Total score	≥13 = indicative of a problematic grief trajectory

### 2.3. Provisional screening tool for problematic grief trajectories

As a next step, we conducted ROC analyses on the sum-score of the four identified early indicators to detect the best cut-off score for our provisional screening tool for problematic grief trajectories. A sum-score ≥ 13 was found to be an optimal cut-off for membership of class 1 or 2 (i.e. persistent high or moderate PGD symptoms). The PPV was 0.84 and the NPV was 0.91 (AUC = 0.91, SE = 0.02, *p *= .00, sensitivity = 0.84, specificity = 0.80, prevalence class 1 = 6%, prevalence class 2 = 35%). A PPV of 0.84 means that a positive result of this test (sum-score of ≥ 13) gives a probability of 0.84 that the individual will develop a problematic grief trajectory. A NPV of 0.91 means that a negative result of this test (sum-score of < 13) gives a probability of 0.91 for the individual to not develop a problematic grief trajectory (Table 5).

## 3. Discussion

We identified four classes of bereaved individuals with a similar trajectory of PGD symptoms. In the previous study of Melhem et al. (2011) in a sample of children, three classes were found. A possible explanation could be that the nature of the sample and losses differed between both studies. Children’s grief reactions are strongly shaped by their developmental capacities and may therefore be expressed differently than adult’s reactions (Christ, Siegel, & Christ, 2002; Miller, 2009). Furthermore, it is known that the cause of death may influence bereavement outcome (Djelantik et al., 2017).

Next, we examined early indicators of the classes with problematic grief trajectories. Experiencing symptoms ‘yearning’, ‘stunned’, ‘life is empty’ and ‘bitterness’ often during the past month at T1 predicted membership of classes with a problematic grief trajectory. The indicators found in our study are different from the symptoms selected in earlier studies examining early indicators of disturbed grief (Guldin, O’Connor, Sokolowski, Jensen, & Vedsted, 2011; Melhem et al., 2013; Shear, Jackson, Essock, Donahue, & Felton, 2006). There could be several reasons for this. Firstly, the symptoms and criteria for a grief disorder are an ongoing subject of debate in scientific publications. All studies used different criteria to select their predictive grief symptoms (Horowitz et al., 2003; Prigerson et al., 1995). We only had data on 11 symptoms included in the PGD scale, while other symptoms like ‘changed world view’ were not included in our analyses. Furthermore, the methodology differed. For instance, in the study of Shear et al. (2006), experts selected the screening items, while in other studies ROC analyses were used.

The following limitations of our study need to be mentioned. Firstly, our study is based on self-report questionnaires. This means that we only could examine the levels of PGD symptoms, instead of PGD diagnoses. Secondly, the classes are based on two time points. To be able to make more detailed trajectories of grief symptoms, more measurement points are needed. Thirdly, our sample was a convenience sample, with a high heterogeneity of losses and an overrepresentation of women. Therefore, generalization should only be done with caution. In the future, predictive symptoms need to be examined in longitudinal studies with more measurement points, whereas PGD diagnosis should be confirmed by clinical interviews.

Notwithstanding these limitations, this study creates more insight into markers of disturbed grief. One could argue that because ‘yearning’ is so commonly experienced by bereaved individuals, it would not be suitable as a predictive symptom for disturbances (Djelantik et al., 2017). However, an often experience of ‘yearning’ was identified as an early indicator of distress. Apparently, it does matter how often and how much time a bereaved individual yearns for his or her loved one in the first year. Surprisingly, the symptom ‘non-acceptance of the loss’ did not have a high sensitivity to predict membership of classes with problematic grief trajectories. However, the specificity of this symptom was high (Tables 3 and 4). So, if bereaved individuals do not accept the loss of their loved one in the first year after the loss, this is not highly predictive for a problematic grief trajectory. Meanwhile, if bereaved individuals accept the loss of their loved one, this is highly predictive for a more favourable course of grief reactions.

In conclusion, this first study about early indicators of problematic grief trajectories among adults will help caregivers to identify bereaved individuals at risk for developing psychopathology. Endorsement of the four early indicators with a cut-off score of ≥ 13 may be used as a screening tool to identify bereaved people at risk for problematic grief trajectories. This generates opportunities to be selective in referring bereaved individuals presenting at victim support organizations or healthcare centres for more extensive diagnostic evaluation and to offer follow-up visits only to those who need them most.
